# Psychiatric Dimensions of Obsessive Behavior: Forensic Insights from Stalking Cases in Serbia

**DOI:** 10.1192/j.eurpsy.2026.11291

**Published:** 2026-07-31

**Authors:** E. Bojovic, J. Sredanovic, M. Stojanovic-Tasic, D. Dimitrijevic, T. Novakovic, D. Stamenkovic, J. Bojovic, J. Tomasevic

**Affiliations:** 1 Clinic for Mental Disorders “Dr Laza Lazarević”, Belgrade; 2 Faculty of Medicine, University of Pristina, Kosovska Mitrovica, Serbia

## Abstract

**Introduction:**

Stalking is a complex and understudied form of interpersonal violence with significant psychiatric and forensic implications. Perpetrators often present with psychotic or delusional disorders, while victims face emotional and physical harm. Limited forensic resources and lingering mental health stigma in Serbia further complicate effective intervention and prevention.

**Objectives:**

Examine the psychiatric characteristics of legally prosecuted stalkers and discuss their relevance for forensic psychiatric practice and preventive strategies.

**Methods:**

A retrospective study was conducted from January 2020 to January 2024, including 46 individuals undergoing court-ordered psychiatric treatment at the Clinic for Mental Disorders “Dr Laza Lazarević” in Belgrade. Data were obtained from medical records and court documentation.

**Results:**

Participants had a mean age of 49.5 ± 12.9 years; males predominated (89.1%). Most were unmarried (91.3%), unemployed (65.2%), and lived with parents (67.4%). The most common diagnoses were delusional disorder (F22; 23.9%) and acute psychotic disorder (F23; 23.9%), followed by unspecified nonorganic psychosis (F29; 21.7%), schizophrenia (F20; 15.1%), organic mental disorders (F06; 6.7%), personality disorders (F60; 4.3%), and bipolar affective disorder (F31; 2.2%). Behavioral patterns differed by diagnosis: F22 cases most often targeted desired partners (33.3%), whereas F23 cases pursued individuals from their social environment (33.3%). Statistical analysis indicated a significant association between unmarried status and social-environment targets. After treatment, 12.8% of participants reoffended, highlighting persistent behavioral risk despite intervention. These findings underscore the heterogeneity of stalking behaviors and the predominance of psychotic-spectrum disorders.

**Conclusions:**

Stalking exemplifies how psychotic-spectrum disorders can escalate into high-risk behaviors. These results highlight the need for culturally adapted, multidisciplinary forensic psychiatric frameworks that integrate clinical care, risk assessment, early intervention, and community-based preventive strategies to protect potential victims and reduce recidivism.

**Disclosure of Interest:**

None Declared

